# A diffusion-based approach for modelling crack tip behaviour under fatigue-oxidation conditions

**DOI:** 10.1007/s10704-018-0311-x

**Published:** 2018-09-06

**Authors:** R. J. Kashinga, L. G. Zhao, V. V. Silberschmidt, R. Jiang, P. A. S. Reed

**Affiliations:** 1 Wolfson School of Mechanical, Electrical and Manufacturing Engineering, Loughborough University, Loughborough, LE11 3TU UK; 2 School of Engineering, The Copperbelt University, Riverside Campus, Jambo Drive, Box 21692, Kitwe, Zambia; 3 Materials Group, Engineering and the Environment, University of Southampton, Southampton, SO17 1BJ UK; 4 College of Energy and Power Engineering, Nanjing University of Aeronautics and Astronautics, Nanjing, 210016 China

**Keywords:** Crack-tip behaviour, Accumulated plastic strain, Oxygen penetration, Finite element, Diffusion analysis

## Abstract

Modelling of crack tip behaviour was carried out for a nickel-based superalloy subjected to high temperature fatigue in a vacuum and air. In a vacuum, crack growth was entirely due to mechanical deformation and thus it was sufficient to use accumulated plastic strain as a criterion. To study the strong effect of oxidation in air, a diffusion-based approach was applied to investigate the full interaction between fatigue and oxygen penetration at a crack tip. Penetration of oxygen into the crack tip induced a local compressive stress due to dilatation effect. An increase in stress intensity factor range or dwell times imposed at peak loads resulted in enhanced accumulation of oxygen at the crack tip. A crack growth criterion based on accumulated levels of oxygen and plastic strain at the crack tip was subsequently developed to predict the crack growth rate under fatigue-oxidation conditions. The predicted crack-growth behaviour compared well with experimental results.

## Introduction

Nickel-based superalloys are designed and optimized for application in hot sections of aero-engines and land-based gas-turbine engines working in harsh conditions. Typical operating conditions combine high static/cyclic loads, high temperatures and non-ambient environments. Highly corrosive exhaust gases attack material surfaces through oxidation, eventually leading to a change in the underlying microstructure and deterioration of material properties. Hence, stability of mechanical properties during service is a critical requirement determined by a material’s resistance to degradation under the attack of environmental constituents. Although nickel-based superalloys are known for their good mechanical properties at high temperatures, inferior behaviour in oxygen-rich environment has been reported, as compared to vacuum conditions, due to oxidation embrittlement, especially the acceleration of crack growth under fatigue-oxidation conditions (Leo Prakash et al. 2009; Bensch et al. 2010; Jiang et al. 2014). At high temperature and in oxygen-rich environments, accelerated crack growth is a consequence of dynamic embrittlement (Pfaendtner and McMahon Jr 2001; Christ et al. 2016) and/or stress-assisted grain boundary oxidation (Molins et al. 1997; Kitaguchi et al. 2013; Jiang and Reed 2016) occurring at a crack-tip subjected to cyclic loading. This is a result of diffusion of oxygen into a crack tip, especially when the crack is fully open (Kitaguchi et al. 2013; Viskari et al. 2013). Diffusion of oxygen into the material, coupled with a counter-diffusion of alloying elements such as Cr, Al and Ni, was reported to initiate changes in microstructure, resulting in inferior load-bearing capability of the material (Bensch et al. 2010, 2013). In addition, the oxides formed at the crack-tip have multi-layered structures similar to those reported in stress-free oxidation studies (Molins et al. 1997), causing material embrittlement.

**Fig. 1:**
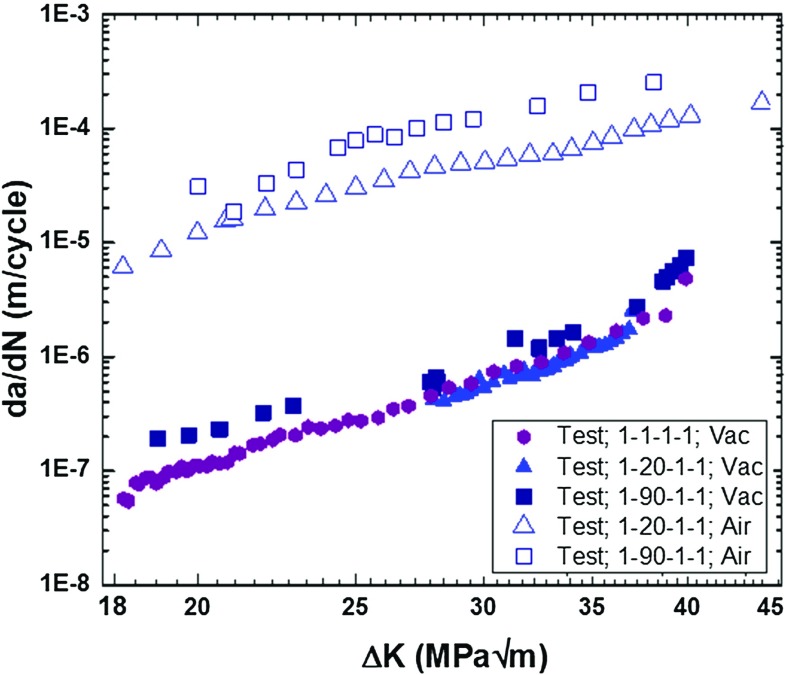
Fatigue crack growth test results in vacuum and air at $725\,^{\circ }\hbox {C}$, showing loading-waveform and environment dependency (Jiang et al. 2017)

Oxidation-accelerated fatigue crack growth (FCG) was studied widely for high-temperature materials. Very recently, the influence of oxidation on crack-growth behaviour in a traditional polycrystalline nickel-based superalloy LSHR (low solvus high refractory; produced through a powder metallurgy route) was studied by Jiang et al. (2017) using single-edge notched bend (SENB) specimens at $725\,^{\circ }\hbox {C}$. For the loading cycles considered, FCG behaviour showed considerable dwell and environment effects as shown in Fig. 1. In a vacuum environment, the specimen exhibited the lowest FCG rate for 1–1–1–1 load waveform (monotonic loading in 1 s, dwell at peak load for 1 s, unloading to minimum load in 1 s and dwell at minimum load for 1 s) and showed the highest rate for 1–90–1–1 waveform (same as 1–1–1–1 but with a dwell of 90 s). As compared to the vacuum environment, tests conducted in air showed two orders of magnitude increase in crack growth rates. This difference is depicted by the correlation between the FCG rate and the levels of stress intensity factor range $(\Delta \hbox {K})$ in Fig. 1. Generally, it is acknowledged that changes in testing environment and loading conditions result in changes in crack growth behaviour (or generally, mechanical behaviour) for most nickel-based superalloys. This has been reported for single-crystal (MacLachlan and Knowles 2001), directionally solidified (DS) (He et al. 2014) and traditional polycrystalline (Jiang et al. 2014) alloys.

Computational models were developed to study oxidation-enhanced FCG behaviour widely observed in nickel-based superalloys. He et al. (2014) predicted enhanced crack growth behaviour, at high temperatures, in a DS nickel-based superalloy, based on the modification of the Paris law. Kiyak et al. (2008) considered material oxidation as the most prevalent on crack flanks, and therefore modelled its influence on FCG behaviour. In the study, time-dependent uniaxial expansion of a layer of finite elements, with a gradual alteration of yield stress, was simulated to reflect the consequence of growth of surface oxides (Kiyak et al. 2008). Although predictions were in agreement with test results, stress-oxidation interaction at a crack-tip was completely neglected. Interaction between oxidation and deformation is key to understand the oxidation-increased FCG. Recent attempts to model this phenomenon demonstrated that the presence of a mechanical load led to increased oxidation damage, reflected in increased oxygen penetration depths (Zhao 2011; Karabela et al. 2013). These observations appeared to agree with experimental studies in terms of the extent to which stress level affects oxidation (Karabela et al. 2011). However, the study was based on a one-way coupling (sequential coupling) approach, as it only considered the effect of stress on oxygen diffusion while neglecting the effect of oxygen diffusion on a stress state. Therefore, there is a lack of comprehensive scheme dealing with the interaction between mechanical loading and oxidation.

In this paper, the diffusion-based computational approach is further developed to study behaviour of a crack tip subjected to fatigue-oxidation conditions. Full interaction between cyclic loading and oxygen diffusion at a crack tip was investigated using the finite element (FE) method. The material studied was a polycrystalline nickel-based superalloy LSHR, thanks to the availability of experimental data as well as a calibrated cyclic viscoplasticity constitutive model (Farukh et al. 2015). Predictions of crack-growth were carried out for both vacuum and air environments and compared with experimental results.

## Modelling of deformation–diffusion interaction

### Viscoplasticity model

In this study, material deformation was described by a viscoplasticity model based on the constitutive equations developed by Chaboche (1989). In this model, both isotropic (*R*) and kinematic $({\varvec{\alpha }})$ hardening variables are considered, during transient and saturated stages of the cyclic response. Within the small strain hypothesis, the strain rate tensor $\dot{{\varvec{\varepsilon }}}$ was additively decomposed into its elastic $(\dot{{\varvec{\varepsilon }}}_{e})$ and inelastic $(\dot{{\varvec{\varepsilon }}}_{p})$ parts:

1 $$\begin{aligned} \dot{{\varvec{\varepsilon }}}=\dot{{\varvec{\varepsilon }}}_{e} +\dot{{\varvec{\varepsilon }}}_{p}. \end{aligned}$$

The elastic strain $\dot{{\varvec{\varepsilon }}}_{e}$ is assumed to follow the Hooke’s law in accordance with the following relation:

2 $$\begin{aligned} {\dot{\varvec{\varepsilon }}_{e}}=\frac{1+\nu }{E}\dot{{\varvec{\sigma }}} -\frac{\nu }{E}(\text {tr}\dot{{\varvec{\sigma }}}){{\varvec{I}}}, \end{aligned}$$

where *E* and $\nu$ are the material’s Young’s modulus and Poisson’s ratio, respectively; ${\varvec{\sigma }}$ and ${{\varvec{I}}}$ are the stress tensor and the unit tensor of the second rank, respectively, and $\hbox {tr}$ denotes the trace.

On the other hand, the viscoplastic (i.e., plastic and creep) strain is represented by the inelastic strain ${\varvec{\varepsilon }}_{p}$. A power-law relationship was adopted for the viscous potential, and the viscoplastic strain rate $\dot{{\varvec{\varepsilon }}}_{p}$ was expressed as (Chaboche 1989):

3 $$\begin{aligned} \dot{\varvec{\varepsilon }}_{p}=\left\langle \frac{f}{Z}\right\rangle ^{n}\frac{\partial f}{\partial {\varvec{\sigma }}}, \end{aligned}$$

where *f* is the von Mises yield function, *Z* and *n* are the material constants, and the Macaulay bracket is defined as

4 $$\begin{aligned} \langle x\rangle =\left\{ \begin{array}{l} x,\quad x\ge 0, \\ 0,\quad x<0. \\ \end{array}\right. \end{aligned}$$

In accordance with the von Mises yield criterion, the yield function *f* was defined as

5 $$\begin{aligned} f({\varvec{\sigma }},{\varvec{\alpha }},R,k) =J({\varvec{\sigma }}-{\varvec{\alpha }})-R-k\le 0, \end{aligned}$$

where ${\varvec{\alpha }}$ is the back stress (the non-linear kinematic hardening variable), *R* is the isotropic hardening variable and *k* is the initial value of the radius of the yield surface. *J* denotes the von Mises distance in the deviatoric-stress space:

6 $$\begin{aligned} J({\varvec{\sigma }}-{\varvec{\alpha }}) =\sqrt{\frac{3}{2} ({\varvec{\sigma }}'-{\varvec{\alpha }}'): ({\varvec{\sigma }}'-{\varvec{\alpha }}')} \end{aligned}$$

where ${\varvec{\sigma }}'$ and ${\varvec{\alpha }}'$ are the deviators of ${\varvec{\sigma }}$ and ${\varvec{\alpha }}$, respectively, (:) denotes the inner product of two tensors. Inelastic flow occurs under the condition $f = 0$ and $\frac{\partial f}{\partial {\varvec{\sigma }}}:{\dot{\varvec{\sigma }}}>0$. For this model, the motion of the yield surface continues to hold but the stress in excess of the yielding point is admissible and often termed as “overstress”.

The evolution of the kinematic stress tensor ${\varvec{\alpha }}$ and the isotropic hardening parameter *R* may be described through the following rules (Chaboche 1989):

7 $$\begin{aligned} \left\{ \begin{array}{l} {\dot{{\varvec{\alpha }}}}={\dot{{\varvec{\alpha }}}}_{1}+{\dot{{\varvec{\alpha }}}}_{2} \\ {\dot{{\varvec{\alpha }}}}_{1}=C_{1}(a_{1}{\dot{{\varvec{\varepsilon }}}}_{p} -{\varvec{\alpha }}_{1}\dot{p})\\ {\dot{{\varvec{\alpha }}}}_{1}=C_{2}(a_{2}{\dot{{\varvec{\varepsilon }}}}_{p} -{\varvec{\alpha }}_{2}\dot{p})\\ \end{array}\right. \qquad \text {and}\qquad \dot{R}= b(Q- R)\dot{p},\nonumber \\ \end{aligned}$$

where $C_1, {a}_{1}, C_{2}, {a}_{2}\,b$ and *Q* are 6 material- and temperature-dependent constants, which determine the shape and amplitude of stress-strain loops during the transient and saturated stages of the cyclic response, and $\dot{p}$ is the accumulated inelastic strain rate defined as:

8 $$\begin{aligned} \dot{p}=\left\langle \frac{f}{Z}\right\rangle ^{n} =\sqrt{\frac{2}{3}~d{{\dot{\varvec{\varepsilon }}}_{p}}: d{{\dot{\varvec{\varepsilon }}}_{p}}}. \end{aligned}$$

Model parameters were calibrated in Farukh et al. (2015) against the data of uniaxial tensile tests for the studied material at $725\,^{\circ }\hbox {C}$. The tests included monotonic tensile tests (strain rate = 0.0083%/s), cyclic tests (strain rate = 1%/s, strain range = 1% and strain ratio = 0) and stress relaxation at 1% strain. The whole process was aimed at obtaining a set of parameters to attain the minimum difference between the test data and the simulation results. Table 1 shows the calibrated parameters. The model was programmed into a user-defined material subroutine (UMAT) using a fully implicit integration (Euler backward iteration algorithm) and implemented in the finite-element software ABAQUS.

**Table 1:** Model parameter values for viscoplastic constitutive model (Farukh et al. 2015)

Parameters	Optimised values
*E* (GPa)	178.77
*b*	6.37
*Q* (MPa)	171.49
$a_{1}$ (MPa)	272.45
$C_{1}$	2123.61
$a_{2}$ (MPa)	306.78
$C_{2}$	2587.69
*Z*	2018.32
*n*	5.17
*k* (MPa)	126.23

### Stress-assisted oxygen diffusion

Stress-assisted oxygen diffusion was modelled by combining two processes. The first process, the flux of oxygen into a metal via natural diffusion is governed by a gradient of the chemical potential at a given state. The second effect was due to application of a mechanical load which was considered to have an influence on the chemical potential. So the governing relationship was obtained as (Larcht’e and Cahn 1982; Stephenson 1988)

9 $$\begin{aligned} \frac{\partial C_{O_2}}{\partial t}=\nabla (D\nabla C_{O_2} -DC_{O_2} M\nabla p), \end{aligned}$$

where $C_{O_2}$ is the oxygen concentration, *t* is the time, *p* is the hydrostatic stress, $\nabla$ stands for the gradient, *D* is the lattice/oxygen diffusion constant (also known as diffusivity) and *M* is the pressure factor, which accounts for the effect of the hydrostatic stress state on diffusion. In Eq. 1, the gradient of the hydrostatic stress is considered to be the driving force for stress-assisted transport of oxygen into the material.

### Diffusion-caused dilatation

It is widely acknowledged (Suo et al. 2003; Elkadiri et al. 2008) that oxidation can generate stresses, which tend to increase as the oxide-scale thickness grows. In particular, the growth of oxides is considered to introduce localised material expansion, leading to generation of dilatational strain. However, the local dilatation is constrained by the surrounding bulk material, hence resulting in a compressive stress (Suo et al. 2003). In fact, this is a process of compositional dilatation caused by oxygen ingress, analogous to thermal expansion in heat transfer. Essentially, assuming isotropic diffusion and expansion, compositional dilatation strain $\varepsilon _{{\mathrm{com}}}$ is given by

10 $$\begin{aligned} \varepsilon _{{\mathrm{com}}}=\beta \Delta C_{O_2}, \end{aligned}$$

where $\beta$ is the dilatational coefficient and $\Delta C_{O_2}$ is the change in oxygen concentration. In this way, the influence of oxidation on crack-tip deformation was taken into account in our numerical simulations.

**Fig. 2:**
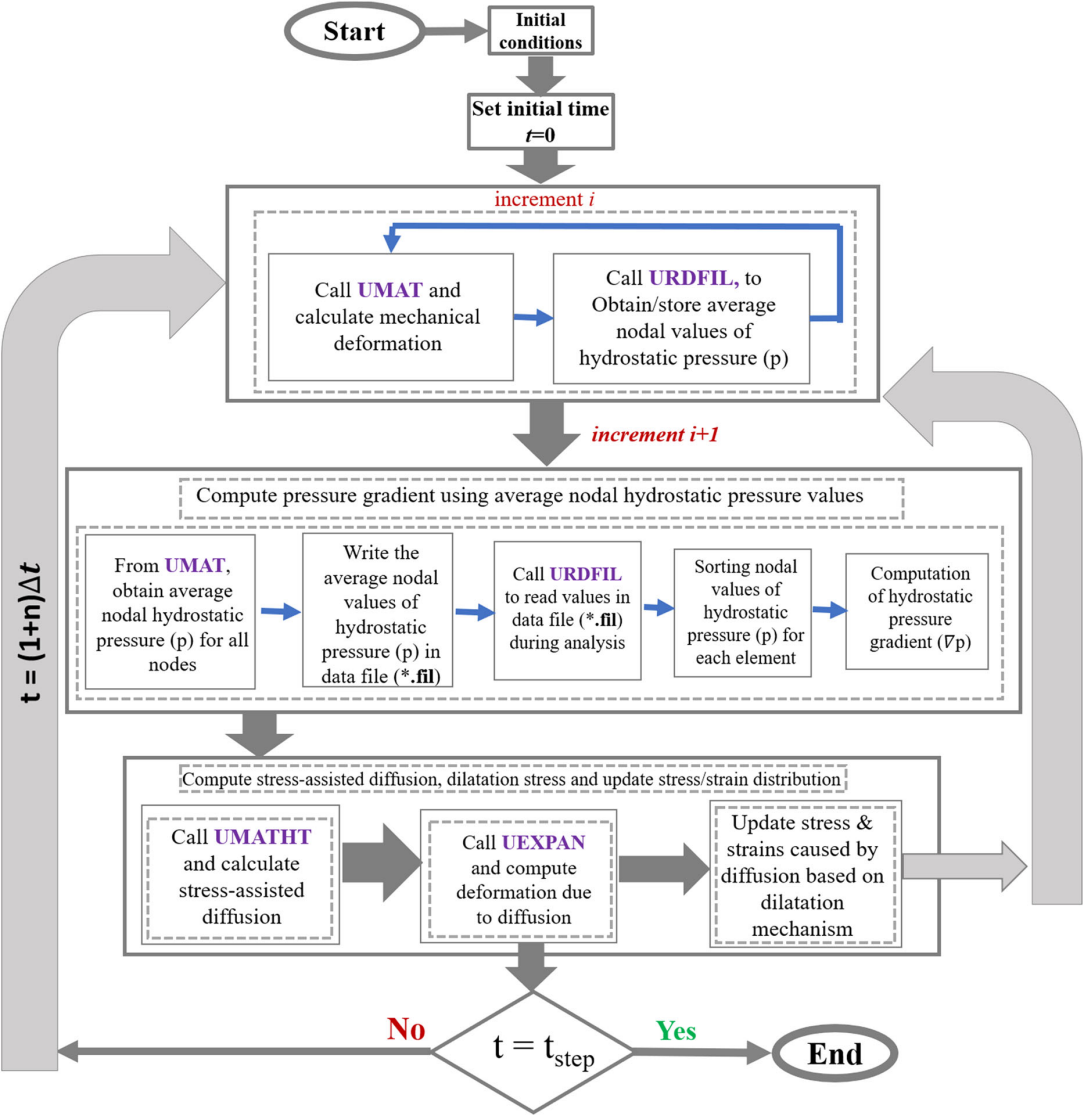
Flow chart outlining the numerical procedure and sequence of the diffusion-based modelling approach via the development of user-defined subroutines UMAT, URDFIL, UMATHT and UEXPAN

### Numerical implementation

Implementation of the interaction between deformation and diffusion analysis was based on a series of subroutines for FE software ABAQUS [(UMAT (user-defined material subroutine), URDFIL, UMATHT and UEXPAN] used to define and compute constitutive behaviour, the gradient of hydrostatic stress, the mass diffusion process and compositional dilatation. Constitutive behaviour of LSHR was described with a cyclic-viscoplasticity model via a user-material subroutine UMAT (Zhao and Tong 2008); the model was calibrated and proven to simulate cyclic deformation and stress relaxation of the material very well (Farukh et al. 2015). Using a subroutine called URDFIL, gradients of hydrostatic stress $(\nabla p)$ were calculated based on a Jacobian transformation matrix linking the global system of coordinates (*x*, *y*) to the local one $(\xi ,\eta )$ in ABAQUS. For diffusion analysis, a subroutine called UMATHT was adopted which utilised the calculated gradient of the hydrostatic stress as input for computation of stress-assisted diffusion. Assessment of dilatation strains was implemented using the subroutine UEXPAN by following Eq. 2, subsequently updating the stress field. Main elements of the overall numerical procedure are outlined in Fig. 2. All the subroutines have variables shared among them which were implemented through solution dependent state variables in ABAQUS. Also, common blocks are used for transferring data between different subroutines.

**Fig. 3:**
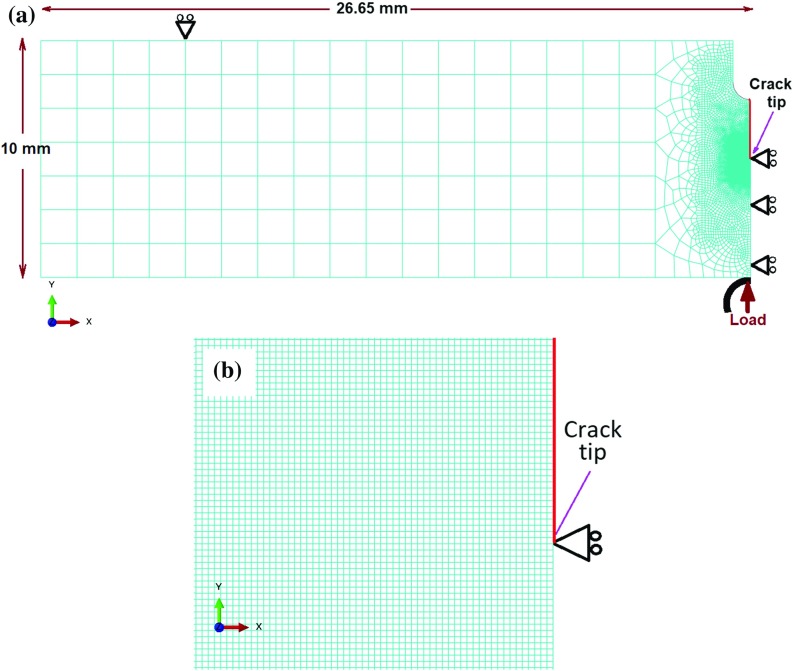
Finite element model for stationary crack analyses: **a** mesh and loading for the half (due to symmetry) SENB specimen and **b** near tip refined mesh with red line showing the pre-crack

It should be noted that the method described above is a continuum modelling approach. Since the alloy is a polycrystalline material, oxygen diffusion along the grain boundaries (GBs) and into the bulk (i.e., grain interior) occurs during the process of oxidation. The continuum model allows us to simulate the process without the need of distinguishing bulk and GB diffusion processes. This significantly reduces the complexity of the problem, given the sophisticated nature of full coupling between viscoplastic deformation and oxygen diffusion near the crack tip. Further research, by coupling crystal plasticity (grain interior), GB deformation and bulk/GB oxygen diffusion, is ongoing in our group and the results will be reported in future publications.

## Finite element model

An SENB specimen geometry used to perform the experimental FCG tests was adopted to develop the FE models. The specimen has a dimension of 53.3 (width) $\times$ 10 (height) $\times$ 10 (thickness) $\hbox {mm}^{3}$, with a notch depth of 2.5 mm in the middle of its width. Thanks to symmetry, only a half model was considered in simulations of deformation behaviour near the stationary crack tip, with plane-strain conditions imposed (Fig. 3). The crack length was chosen to be $a=4.0\,\hbox {mm}$, i.e., $\frac{a}{W}=0.4$, combining the notch (2.5 mm) and the pre-crack (1.5 mm) depths. Trapezoidal loading waveforms of a 1–X–1–1 type (where X is the dwell time at the peak load, and X = 1, 20 and 90 s) were applied, with a load ratio $R_L =0.1$. Three load levels, i.e., 2.185, 3.275 and 4.375 kN, were considered (only half of the load was applied due to the half-model geometry), corresponding to stress intensity factor ranges of $20, 30\,\hbox {and}\,40\,\hbox {MPa}\surd \hbox {m}$, respectively. To avoid a rigid-body motion, the support at the top surface of the model was prevented from movement in the loading direction (Fig. 3a). Since only one half of the SENB specimen was considered in the model, additional symmetry constraint was implemented along the middle line in the *x*-direction (Fig. 3a.). Cyclic mechanical loading was applied to the middle point of the SENB specimen, through a rigid pin as shown in Fig. 3a.

To reduce computational costs, a fine mesh with an element size of $5\,{\upmu }\hbox {m}$ was only applied in the near-tip region (Fig. 3b), while a coarse mesh was applied elsewhere (Fig. 3a). The average grain size of the material is $36.05 \pm 18.07\,{\upmu }\hbox {m}$ (Jiang et al. 2014), several times the mesh size $(5\,{\upmu }\hbox {m})$ near the crack tip. The choice of this mesh size was based on a mesh sensitivity study of the normal strain distribution ahead of the crack tip (less than 5% difference when compared to a mesh with element size of $2.5\,{\upmu }\hbox {m}$ near the crack tip). Considering the balance between computational efficiency and mesh convergence, the mesh with an element size of $5\,{\upmu }\hbox {m}$ near the crack tip was adopted in this study (Fig. 3). A total of 12,495 four-node quadrilateral CPE4T elements with full integration were generated. These were plane strain elements with temperature (i.e., oxygen concentration) as an additional degree of freedom. In order to maintain the accuracy of simulation results, full integration has been adopted, allowing four Gauss integration points for each element. If the reduced integration was used, only one integration point would be considered for each element, compromising the accuracy of FE simulations. As the viscoplasticity model is within a small deformation regime, full integration does not impose any convergence issue on the simulations. An initial oxygen concentration of zero was assigned for the model. A flux boundary condition (Zhao 2011) was applied to a crack surface in the diffusion analysis based on a parabolic-rate constant $(\kappa _{{{\varvec{p}}}})$ determined from thermogravimetric analysis of this material under oxidation (see Sect. 4.2). This flux boundary condition was implemented in ABAQUS using the subroutine DFLUX.

## Results and discussion

### Crack-tip behaviour and crack growth in vacuum

**Fig. 4:**
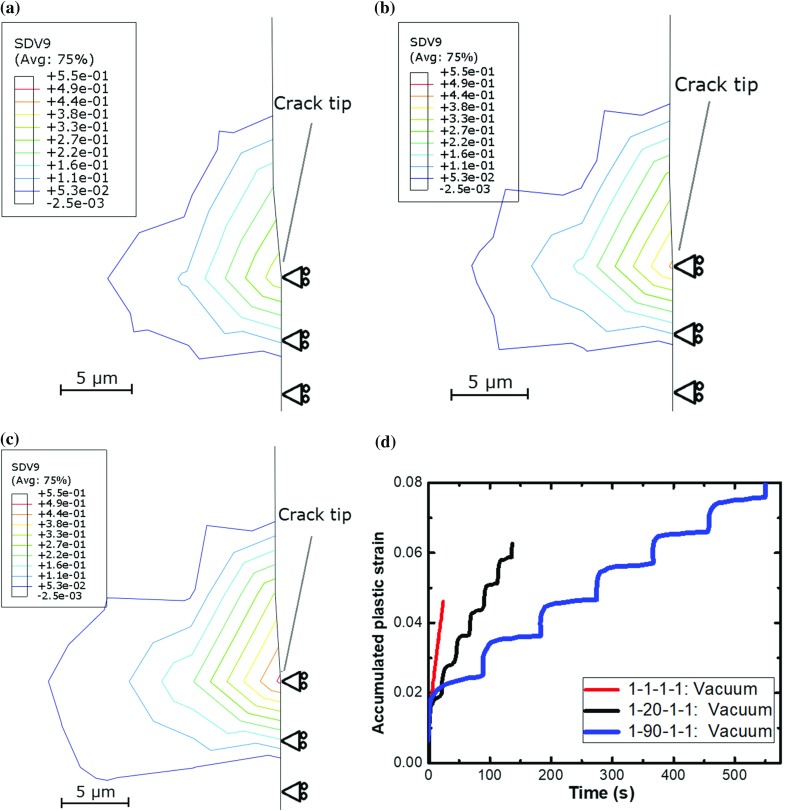
Contour plots of the accumulated plastic strain for **a** 1–1–1–1, **b** 1–20–1–1 and **c** 1–90–1–1 waveforms at the end of 10 loading cycles. Its evolution with loading times is shown in **d** for the element just ahead of the crack tip

For the three trapezoidal loading waveforms (1–1–1–1, 1–20–1–1 and 1–90–1–1) with load ratio $R_L =0.1$, deformation processes near the crack tip were studied for three ranges of stress intensity factor. Particularly, material damage due to fatigue loading was assessed by a solution-dependent variable (SDV), accumulated plastic strain, widely used in reflecting fatigue damage (Zhao and Tong 2008; Bower 2009). After 15 loading cycles, different levels of damage were observed for the three loading regimes considered, as reflected by contour plots of accumulated plastic strain (SDV9; Fig. 4). It can be seen that at $\Delta K= 30\,\hbox {MPa}\surd \hbox {m}$, the damage accumulation was highly dependent on the loading waveforms. The highest level of fatigue damage was found in the specimen subjected to 1–90–1–1 loading (see Fig. 4c) while 1–1–1–1 resulted in the lowest level (see Fig. 4a). Furthermore, the accumulated plastic strain was monitored over the time at the centroid of the element just ahead of the crack tip (Fig. 4d), which has the most accumulated plastic strain and is directly associated with crack growth. It reveals an increase in the accumulation of damage in all the three cases; however, the specimen subjected to the 1–90–1–1 loading waveform exhibited the highest rate of damage accumulation. This could be the reason for the highest crack growth rate observed for this loading regime in experiments (see Fig. 1). The slope variation of accumulated plastic strain within each fatigue cycle is as expected. This is due to the difference in plastic deformation experienced by the material during the loading stage, the first dwell (peak load), the unloading stage and the second dwell (minimal load) within each fatigue cycle. The specimen was subjected to load-controlled, instead of strain-controlled, loading conditions, so strain accumulation during the load-hold times was a result of creep deformation (viscoplastic behaviour) of the material. The plastic deformation, caused by creep at dwell, showed a primary stage (rapid increase) and a secondary stage (steady state), which corresponded to different rates of plastic strain accumulation (i.e., slope variation).

Additionally, an extended finite-element method (X-FEM) was used to predict fatigue-crack propagation under vacuum conditions. X-FEM was chosen in preference to other approaches such as cohesive element and virtual crack-closure techniques, due to its capability of predicting a path and a rate of propagation of a fatigue crack based on a solution-dependent criterion (Karihaloo and Xiao 2003; Abdelaziz and Hamouine 2008), without pre-defining the path. By incorporating an enrichment function accounting for field discontinuities, X-FEM makes it possible for the crack to propagate through arbitrary elements, eliminating the need for remeshing throughout the analysis. This is mainly achieved by enriching a classical displacement-based FE approximation for the crack according to a partition of unit (Karihaloo and Xiao 2003; Abdelaziz and Hamouine 2008). In this part of the study, predictions were carried out using a full-geometry FE model consisting of four-node plane-strain elements with full integration. Within a crack-growth region, structured elements of $5\,{\upmu }\hbox {m}$ size were used; this mesh size was fine enough to capture adequately a crack-tip cyclic plastic zone (Huseyin and Wei 1989; Zhao et al. 2010). An initial crack length of $3.775\,\hbox {mm}$, combining the notch (2.5 mm) and pre-crack (1.275 mm), was considered; which corresponded to the experimental FCG tests.

**Fig. 5:**
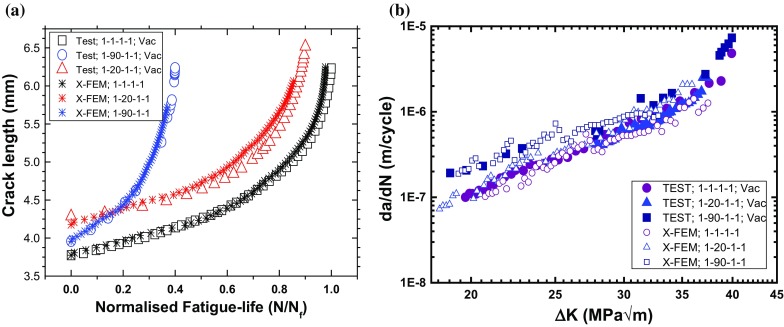
Prediction of FCG in LSHR by X-FEM at $725\,^{\circ }\hbox {C}$ in a vacuum: **a** crack length against a normalised number of cycles, **b** correlation between FCG rate and stress intensity factor range

Suitability of accumulated plastic strain to describe damage under cyclic loading (see Fig. 4d) (Zhao and Tong 2008; Bower 2009) led to its employment as a criterion to predict FCG in vacuum. Specifically, the onset of crack growth was controlled by a solution-dependent variable indicating the accumulation of plastic strain. A user-defined subroutine UDMGINI, in combination with the main user-defined material subroutine (UMAT), was used to assess material damage at the crack tip. A threshold value of 8.8% for the accumulated plastic strain was used to trigger the onset of crack growth for all the loading conditions considered. Crack growth rates were determined by monitoring the crack length over the number of cycles and correlated with $\Delta K$. As shown in Fig. 5, XFEM predictions are in good agreement with the test data for both the crack length and the crack growth rate, including the loading-waveform dependency.

**Fig. 6:**
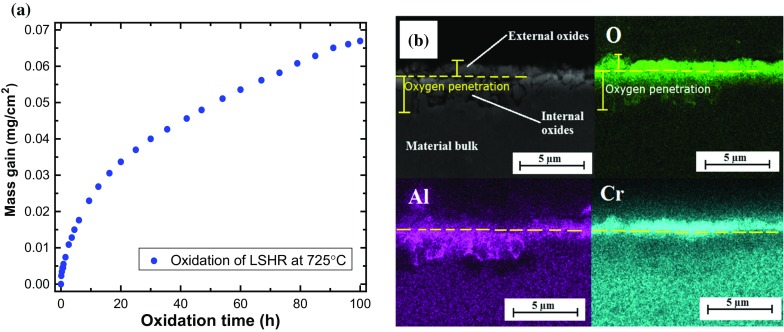
Oxidation behavior of LSHR in laboratory air at $725^{\circ }\hbox {C}$ (for 100 h): **a** mass gain during oxidation, **b** cross-sectional analysis by SEM and EDS with element maps for oxygen (O), aluminium (Al) and chromium (Cr)

**Table 2:** Parameters used in diffusion analysis

Parameter	Value
Diffusivity (*D*)	$3.403 \times 10^{-11}\, (\hbox {mm}^{2}/\hbox {s})$
Oxidation parabolic rate constant $({\upkappa }_{p})$	$1.236 \times 10^{-10}\,(\hbox {g}^{2}/\hbox {mm}^{4}/\hbox {s})$
Dilatational coefficient $(\beta )$	$2.475 \times 10^{-2 }\,(\hbox {mm}^{3}/\hbox {g})$
Pressure factor (*M*)	$9.500 \times 10^{-2}\,(\hbox {MPa}^{-1})$

### Calibration of diffusion parameters

The four parameters required for diffusion-based analysis include the oxygen diffusivity (*D*), the pressure factor (*M*), the dilatation coefficient $(\beta )$ and the parabolic oxide growth constant $({\upkappa }_{p})$. To obtain ${\upkappa }_{p}$ for the studied material, static oxidation tests (stress-free tests) were conducted in laboratory air for 100 h using a thermogravemtric analysis (TGA) rig (Setaram *Setsys Evaluation* furnace). The rig has the capability to measure the mass gain at temperatures up to about $2400\,^{\circ }\hbox {C}$ in controlled environments, with a precision of $10\,{\upmu }\hbox {g}$. Disc specimens, 10 mm in diameter and 1.5 mm in thickness, were subjected to isothermal conditions at $725\,^{\circ }\hbox {C}$ ($\pm \,1.5\,^{\circ }\hbox {C}$ tolerance), with the mass gain recorded at every of 72 s. The measured mass gain per specimen surface area $(\Delta \mathrm{m} \hbox {--} \mathrm{mg/mm^{2}})$ is plotted in Fig. 6a against the oxidation time, which follows a parabolic relationship (Encinas-Oropesa et al. 2008):

11 $$\begin{aligned} (\Delta m)^{2}=\kappa _{p} t, \end{aligned}$$

where *t* is the time. Based on the measurements in Fig. 6a and Eq. (11), $\kappa _{{{\varvec{p}}}}$ was found to be $1.236\times 10^{-10}\hbox {g}^{2}/\hbox {mm}^{4}/\hbox {s}$ for LSHR (see Table 2).

**Fig. 7:**
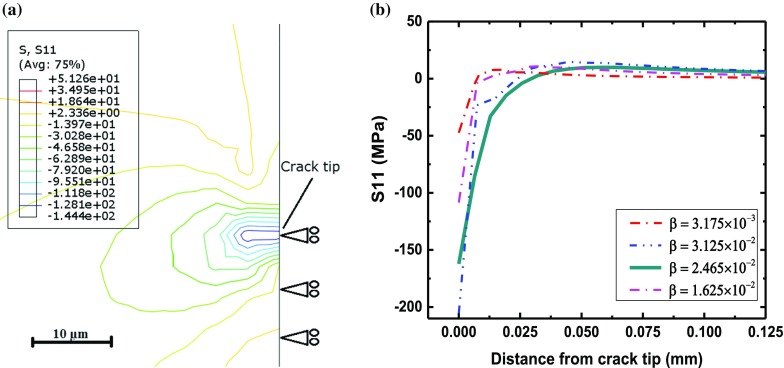
Effect of dilatation coefficient $\beta$ on near-tip stress field: **a** contour plot of the stress (in MPa) normal to the crack plane and **b** variation of the stress normal to the crack plane with distance from the crack tip

In addition, SEM and EDS were used to characterise the cross section of the oxidised disc after sectioning, mounting and polishing the samples. It can be seen, from the SEM micrograph and the element maps in Fig. 6b, that oxygen penetrated the material to form oxides rich in Al and Cr, with the former presented predominantly internally and the latter mostly on external surfaces. Direct measurement of oxygen diffusivity (*D*) was not possible in our study, thus it was estimated from the following relationship (Azari et al. 2008; Karabela et al. 2013):

12 $$\begin{aligned} X^{2}=Dt, \end{aligned}$$

where *X* is the depth of internal oxidation. According to the SEM and EDS analysis results, shown in Fig. 6b, the depth of internal oxidation (oxygen penetration) was about $3.5\,{\mathrm{\mu }}\hbox {m}$ over an oxidation time of 100 h. Based on Eq. (12), the diffusivity was therefore estimated to be $3.403\times 10^{-11}\,\hbox {mm}^{2}/\hbox {s}$ (see Table 2).

The pressure factor was taken from our previous study, where the influence of applied stress on oxidation was investigated for a similar PM nickel-based superalloy (Karabela et al. 2013). It revealed (through FIB measurements) that application of a cyclic stress led to deeper oxygen penetration, as compared to natural diffusion. FE simulations were then carried out, based on Eq. (9), to calibrate the pressure factor by matching the simulated oxygen penetration with FIB measurements over a range of temperatures. For the temperature $(725\,^{\circ }\hbox {C})$ considered in this study, the pressure factor was found to be $9.5\times 10^{-2}\,\hbox {MPa}^{-1}$ (given in Table 2) based on the results in Karabela et al. (2013).

**Fig. 8:**
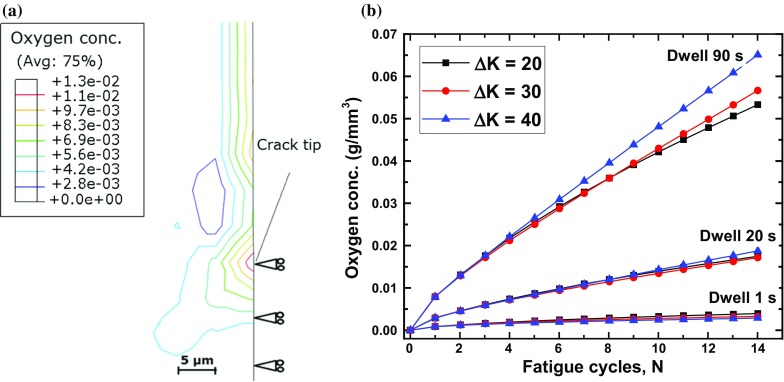
**a** Contour plot of oxygen concentration near the crack tip for 1–20–1–1 waveform with $\Delta K=20\,\hbox {MPa}\surd \hbox {m}$, and **b** effect of stress intensity factor range and dwell times on oxygen concentration at a crack tip

### Effect of oxygen diffusion on crack-tip stress field

Oxygen diffusion into a crack tip causes material expansion and associated dilatation strain, leading to generation of compressive stress. Based on Eq. (10), dilatational strain is controlled by the dilatation coefficient $(\beta )$, which is analogous to a coefficient of thermal expansion. In physical terms, this parameter describes the effect of oxygen on deformation. It was calibrated based on findings of Suo et al. (2003), who studied the effects of (non-reciprocal) diffusion of oxygen and alloy elements during static oxidation. Simulations to calibrate the parameter $\beta$ were therefore carried out by considering dilatation due to natural diffusion of oxygen into the crack tip. This was achieved by setting the load to zero in the model (in Fig. 3); compressive stress resulting from the dilatation strain (due to natural diffusion of oxygen into the crack tip) was then calculated based on the user-subroutine UEXPAN. The simulated stress (normal to the crack plane) due to natural diffusion into the crack-tip was assessed and results are presented in Fig. 7, for a range of $\beta$ values. Apparently, compressive stresses were generated near the crack tip and their magnitude decreased with increasing distance from the crack tip. According to Fig.7b, $\beta =2.465\times 10^{-2}\,\hbox {mm}^{3}/\hbox {g}$ was found to yield a compressive stress of about 165 MPa, as reported in Suo et al. (2003). Consequently, this value of $\beta$ was used to simulate diffusion-deformation interaction at a crack tip in this study.

We also monitored the evolution of accumulated plastic strain near the crack tip, and it was found out that the effect of oxygen diffusion on plastic deformation near the crack tip is almost negligible due to a relatively low value of the dilatation parameter used in the simulations (i.e., $\beta =2.465\times 10^{-2}\,\hbox {mm}^{3}/\hbox {g}$). Specifically, the near-tip stresses caused by oxygen diffusion are typically less than 200 MPa for the loading waveforms considered here, which are negligible when compared to the stresses caused by mechanical loading (typically $>2000\,\hbox {MPa}$ for the loading conditions considered in this study). Consequently, the effect of oxygen diffusion on mechanical deformation near the crack tip is almost negligible for the simulations carried out in this study.

### Oxygen penetration near crack tip

The effect of fatigue loading on oxidation process was studied in terms of oxygen penetration into the crack tip. FE simulations were carried out for selected loading conditions, from which evolution of the oxygen concentration was extracted for a centroid of the element just ahead of the crack tip. The influence of stress intensity factor range and the dwell time on oxygen diffusion near the crack tip is particularly studied in this section.

#### Effects of stress-intensity-factor range

Here, evolution of oxygen concentration was investigated by keeping loading waveform unchanged while increasing the $\Delta K$ level. The results obtained are shown in Fig. 8 in terms of the oxygen concentration against the number of cycles. For 15 load cycles, an increase in the stress-intensity-factor range led, generally, to increased oxygen concentration near the crack tip. For 1–20–1–1 and 1–90–1–1 loading waveforms, the attained magnitudes were considerably higher due to the longer dwell periods. So, under loading conditions with higher dwell times, failure is more likely to be caused by a combination of mechanical deformation and oxidation damage, a common feature for alloys at high temperature (Everitt et al. 2008).

#### Effect of dwell times

The influence of dwell times imposed at peak loads on the penetration of oxygen into a crack tip is shown in Fig. 8 for selected load levels. Apparently, oxygen concentration at the crack tip was the lowest in the specimen subjected to the loading waveform 1–1–1–1 and the highest for 1–90–1–1. This is because the crack remained fully open for the longest period of time (90 s) in each cycle, allowing a larger amount of oxygen to penetrate into the crack tip. At all the studied load levels, a progressive increase in oxygen concentration was observed over the increasing number of loading cycles.

### Crack growth under fatigue-oxidation

**Fig. 9:**
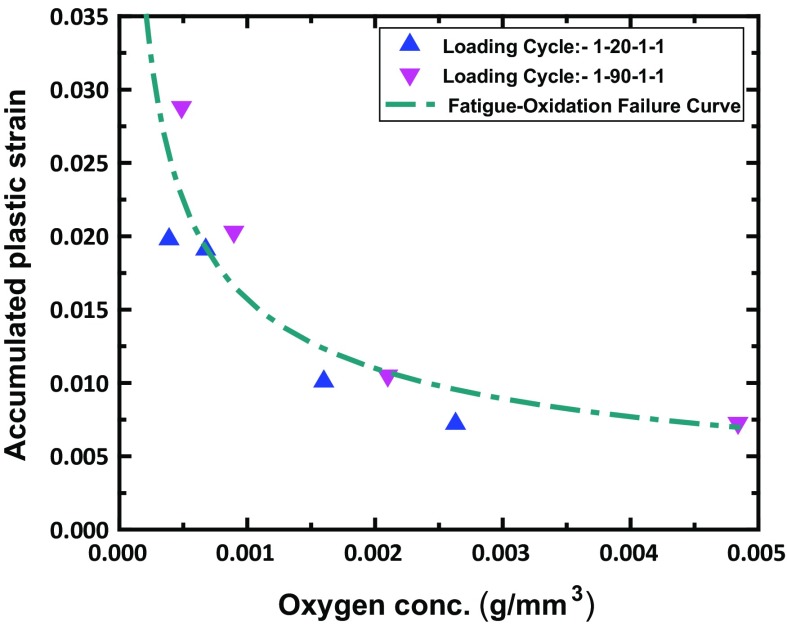
Failure curve expressed in terms of accumulated plastic strain and oxygen concentration at a crack tip. Based on crack growth test data in vacuum and air for 1–20–1–1 and 1–90–1–1 waveforms with $\Delta K=18,20,30\,\hbox {and}\,40\,\hbox {MPa}\,\surd \hbox {m}$

Prediction of crack growth under fatigue-oxidation conditions needs consideration of damage due to both mechanical deformation and oxidation embrittlement. For damage caused by mechanical deformation, the accumulated plastic strain (also used in the X-FEM-based prediction of FCG in vacuum; see Sect. 4.1) was adopted. In order to consider the oxidation effect, oxygen concentration near the crack tip was taken as an additional parameter in this study. In this case, a two-parameter criterion, expressed as a failure curve, was used to predict the onset of the crack growth. This failure curve was constructed from FE simulations based on the crack growth test data in air, for which loading waveforms 1–20–1–1 and 1–90–1–1 at four levels of $\Delta K$ were considered. Specifically, the number of cycles required for the crack to grow (by one element) was calculated by dividing the element size $(5\,\mu \hbox {m})$ with the crack growth rate at the respective $\Delta K$ level (i.e., $18, 20, 30\,\hbox {and}\,40\,\hbox {MPa}\,\surd \hbox {m}$). Diffusion-based FE simulations were then implemented for the calculated number of cycles, and the values of the two damage parameters were extracted at the centroid of the element just ahead of the crack tip. These values were then plotted against each other to form a fatigue-oxidation failure curve as shown in Fig. 9.

**Fig. 10:**
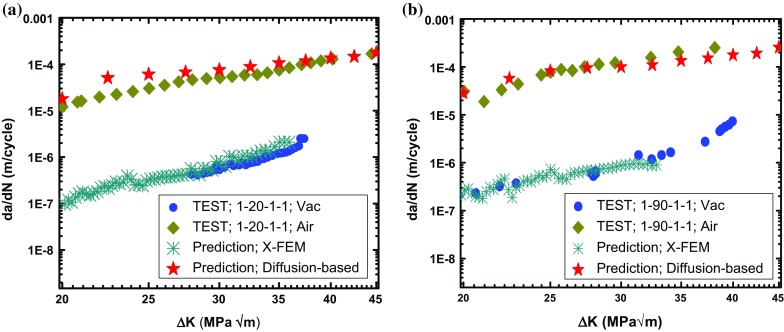
Predicted FCG rates from X-FEM and diffusion-based approach for **a** 1–20–1–1 and **b** 1–90–1–1 loading waveforms, in comparison with test data

Evolution of the two damage parameters with fatigue loading cycles was then modelled with the diffusion-based finite element analyses for a full range of $\Delta K$ levels (from 20 to 50 $\hbox {MPa}\surd \hbox {m}$); both of them were found to increase progressively with the number of loading cycles. These results were then used in conjunction with the failure curve (Fig. 9) to predict the crack growth rate, i.e., to determine the number of cycles required to reach the failure curve, accounting for both the accumulated plastic strain and oxygen concentration near the crack tip. Comparison of predicted and measured crack growth rates is given in Fig. 10 for the loading waveforms 1–20–1–1 and 1–90–1–1. Predictions were in good agreement with test data over a range of the studied $\Delta K$ levels. So, the two-parameter criterion is able to capture the accelerated crack growth under fatigue-oxidation conditions for varied dwell times. It should be noted that the failure curve in Fig. 9 was constructed using the crack growth data at four selected $\Delta \hbox {K}$ levels only (e.g., 18, 20, 30 and $40\,\hbox {MPa}\surd \hbox {m}$). The failure curve was then used to predict the crack growth rates for a whole range of $\Delta \hbox {K}$ levels, i.e., varying between 20 and $50\,\hbox {MPa}\surd \hbox {m}$. Therefore, the results in Fig. 10 are mostly predictions based on the obtained fatigue-oxidation failure curve in Fig. 9.

As oxidation process is essentially controlled by the exposure times, dwell times play an important role for crack extension in oxidation environment. If longer dwell is imposed, oxidation damage will become more significant and crack will grow faster due to accelerated process of material embrittlement. This has been predicted by our simulations. However, this does not diminish the role of fatigue (i.e., no dwell), as accumulated plastic deformation is also largely contributed by the fatigue process. So crack growth under fatigue-oxidation condition is controlled by deformation as well as oxidation, which are both important. Certainly, longer dwell times will accelerate oxidation damage as well as introduce more plastic deformation, leading to faster crack growth rate (in terms of number of cycles).

## Conclusions

Crack-tip behaviour in a nickel-based superalloy LSHR was studied under fatigue-oxidation conditions. In a vacuum, this behaviour is controlled by mechanical deformation, and predictions for the crack growth were based solely on accumulated plastic strain caused by fatigue. Using the extended finite-element method, the effects of loading waveform and stress intensity factor range were successfully predicted. A diffusion-based FE model was developed and applied to predict high-temperature crack-growth behaviour in air for LSHR. In this model, mechanical deformation was described by the cyclic viscoplasticity model while oxidation was described according to mass diffusion. Two parameters, i.e., the accumulated plastic strain and the oxygen concentration, were considered to describe the damage. FE analyses showed that crack tip deformation behaviour was characterised by progressive increases in accumulated plastic strain and oxygen concentration with increasing fatigue loading cycles. Oxygen concentration was found more sensitive to variations in imposed dwell while accumulated plastic strain was sensitive to $\Delta K$ level as well as dwell times. The model has been applied to predict the effects of dwell times imposed at peak loads and the level of $\Delta K$ on crack growth behaviour under fatigue-oxidation conditions.
